# The relationship between weight-adjusted-waist index and diabetic kidney disease in patients with type 2 diabetes mellitus

**DOI:** 10.3389/fendo.2024.1345411

**Published:** 2024-03-15

**Authors:** Zhaoxiang Wang, Xuejing Shao, Wei Xu, Bingshuang Xue, Shao Zhong, Qichao Yang

**Affiliations:** ^1^ Department of Endocrinology, Affiliated Kunshan Hospital of Jiangsu University, Kunshan, Jiangsu, China; ^2^ Department of Endocrinology, Affiliated Wujin Hospital of Jiangsu University, Changzhou, Jiangsu, China; ^3^ Department of Endocrinology, Wujin Clinical College of Xuzhou Medical University, Changzhou, Jiangsu, China; ^4^ Department of Nephrology, Affiliated Wujin Hospital of Jiangsu University, Changzhou, Jiangsu, China

**Keywords:** DKD, obesity, weight-adjusted-waist index, NHANES, population-based study

## Abstract

**Purpose:**

Obesity, particularly abdominal obesity, is seen as a risk factor for diabetic complications. The weight-adjusted-waist index (WWI) is a recently developed index for measuring adiposity. Our goal was to uncover the potential correlation between the WWI index and diabetic kidney disease (DKD) risk.

**Methods:**

This cross-sectional study included adults with type 2 diabetes mellitus (T2DM) who participated in the NHANES database (2007-2018). The WWI index was calculated as waist circumference (WC, cm) divided by the square root of weight (kg). DKD was diagnosed based on impaired estimated glomerular filtration rate (eGFR<60 mL/min/1.73m^2^), albuminuria (urinary albumin to urinary creatinine ratio>30 mg/g), or both in T2DM patients. The independent relationship between WWI index and DKD risk was evaluated.

**Results:**

A total of 5,028 participants with T2DM were included, with an average WWI index of 11.61 ± 0.02. As the quartile range of the WWI index increased, the prevalence of DKD gradually increased (26.76% vs. 32.63% vs. 39.06% vs. 42.96%, *P*<0.001). After adjusting for various confounding factors, the WWI index was independently associated with DKD risk (OR=1.32, 95%CI:1.12-1.56, *P*<0.001). The area under the ROC curve (AUC) of the WWI index was higher than that of body mass index (BMI, kg/m^2^) and WC. Subgroup analysis suggested that the relationship between the WWI index and DKD risk was of greater concern in patients over 60 years old and those with cardiovascular disease.

**Conclusions:**

Our findings suggest that higher WWI levels are linked to DKD in T2DM patients. The WWI index could be a cost-effective and simple way to detect DKD, but further prospective studies are needed to confirm this.

## 1 Introduction

DKD, a significant complication arising from diabetes, is typified by the presence of albuminuria and a declining glomerular filtration rate (GFR) (1). Standing as one of the most rapidly escalating contributors to chronic kidney disease (CKD), DKD is responsible for over half of the cases leading to end-stage kidney disease (ESKD) and associated mortality. In the United States alone, DKD affects upwards of 40% of individuals diagnosed with T2DM, levying a considerable economic and healthcare toll on society (2).

Obesity, particularly abdominal obesity, has long been recognized as a harbinger of diabetic complications (3). In clinical assessment, several common anthropometric measures were used to evaluate obesity and its associated risks, each with inherent limitations. BMI is a prevalent tool but cannot distinguish fat type or distribution, as well as muscle mass (4). WC also poorly predicts visceral adipose tissue at an individual level (4). The “obesity paradox” has been observed to varying degrees, with inconsistent or contradictory results between these obesity indicators in various populations (5). Thus, proposed by Park et al. in 2018, the WWI index, defined as WC divided by the square root of body weight, emerges as a novel obesity metric (6). It capitalizes on the advantages of WC while reducing its correlation with BMI (7). The WWI offers a more nuanced distinction between fat and muscle mass, focusing primarily on abdominal obesity independent of body weight (7, 8). Additionally, emerging evidence has substantiated the notion that an elevation in the WWI index poses a substantial risk factor for metabolic disorders, including diabetes and non-alcoholic fatty liver disease (8–11). Consequently, it is of paramount importance to delve deeper into its correlation with DKD within the diabetic population.

Numerous epidemiological studies highlight the significant role of obesity, especially abdominal obesity, in DKD (3). However, to our knowledge, the role of WWI index in T2DM patients with DKD remains unclear. This study, drawing on the National Health and Nutrition Examination Survey (NHANES) database, sought to explore the association between the WWI index and the prevalence of DKD in a nationally representative sample.

## 2 Materials and methods

### 2.1 Data source

Data utilized in this population-based study was from NHANES, a nationwide survey conducted by the National Center for Health Statistics of the Centers for Disease Control and Prevention, employing a randomized, stratified, multi-stage approach (12). Participants underwent a series of evaluations including physical examinations, health and nutrition questionnaires, and laboratory assessments. The NHANES study protocol was approved by the Ethics Review Board of the National Center for Health Statistics. Comprehensive detailed design and data can be accessed at https://www.cdc.gov/nchs/nhanes/. Six NHANES cycles data were obtained and merged: 2007–2008, 2009–2010, 2011–2012, 2013-2014, 2015-2016, and 2017-2018 (59,842 participants). Participants under the age of 20, pregnant, as well as those without data on WWI index, urinary albumin to urinary creatinine ratio (UACR, mg/g), eGFR, and T2DM were excluded. Finally, a total of 5,028 eligible T2DM patients were included in the study.

### 2.2 Exposure and outcome definitions

The WWI index, as an exposure variable, was defined as WC divided by the square root of weight. Diabetes was defined as either self-reported doctor diagnosis of diabetes, or fasting plasma glucose (FPG, mmol/L) ≥7.0 mmol/L, or glycohemoglobin≥6.5%, or the use of diabetes medication. The urinary albumin to urinary creatinine ratio was used to calculate the UACR, while the eGFR was calculated using the CKD-EPI creatinine equation, considering factors such as age, gender, race, and serum creatinine (Scr, μmol/L) (13). DKD in T2DM patients was diagnosed if UACR was >30 mg/g and/or eGFR was <60 mL/min/1.73m^2^.

### 2.3 Covariate definitions

We collected demographic data including age, gender, and race, along with potential covariates such as annual household income, educational level, physical activity, smoking status, hypertension, cardiovascular disease, BMI, alanine transaminase (ALT, U/L), aspartate transaminase (AST, U/L), gamma-glutamyl transferase (GGT, U/L), FPG, glycohemoglobin, triglycerides (TG, mmol/L), total cholesterol (TC, mmol/L), high-density lipoprotein cholesterol (HDL-c, mmol/L), low-density lipoprotein cholesterol (LDL-c, mmol/L), blood urea nitrogen (BUN, mmol/L), serum uric acid (SUA, umol/L) and Scr. BMI was categorized as <25, 25-29.9, and ≥30 kg/m^2^ representing normal weight, overweight, and obesity. Smokers were identified as current and former. Self-reported hypertension was also defined. The presence of cardiovascular disease was identified based on self-reported history of specific conditions such as heart attack, stroke, congestive heart failure, coronary artery disease, or angina. Detailed measurement procedures for all variables are available in the NHANES database at https://www.cdc.gov/nchs/nhanes/.

### 2.4 Statistical analysis

Population-weighted descriptive statistics was calculated in this study. Continuous variables were reported as mean with standard error (SE), and categorical variables as percentage with SE. Weighted Student’s t-test and chi-squared test were used to compare variables among groups. Weighted Pearson correlation analysis assessed the association of WWI index with other covariates. Logistic regression models were used to investigate associations between weight, WC, WWI index and DKD, low-eGFR (eGFR<60 mL/min/1.73m^2^), and albuminuria (UACR>30mg/g) risk, with three models used for different levels of covariate adjustment (Model 1, without covariates adjusted; Model 2, with adjustments for age, gender, and race; Model 3, with adjustments for multiple covariates including age, gender, race, annual household income, education level, physical activity, smokers, hypertension, cardiovascular disease, BMI, ALT, AST, GGT, FPG, glycohemoglobin, TG, TC, HDL-c, LDL-c, BUN, SUA, and Scr). Smooth curve fitting analysis was used based on a generalized additive model for DKD, low-eGFR, and albuminuria, with WWI index as the independent variable. ROC analysis was used to evaluate the impact of WWI index on DKD, low-eGFR, and albuminuria risk. Subgroup analyses were performed based on age (<60/≥60 years), gender (female/male), race (white/non-white), smokers (yes/no), BMI (normal weight/overweight/obese), hypertension (yes/no), and cardiovascular disease (yes/no). All statistical analyses followed Centers for Disease Control and Prevention guidelines, incorporating a complex multistage cluster survey design, and combining subsample weights from six cycles. The statistical analyses were performed by the Empower software (http://www.empowerstats.com) and R 4.2.1 (http://www.R-project.org). A two-side *P* value <0.05 was considered statistically significant.

## 3 Results

### 3.1 Basic characteristics of T2DM participants

The study involved 5,028 T2DM participants meeting the inclusion criteria, with an average age of 58.89 ± 0.29 years and DKD composition of 38.60%. We analyzed and compared general information and clinical indicators between the non-DKD and DKD groups (**Table 1**). Our results revealed that the DKD group had significantly higher values for age, annual household income under $20,000, hypertension, cardiovascular disease, WC, GGT, glycohemoglobin, FPG, TG, BUN, SUA, Scr, urinary albumin and UACR compared to the non-DKD group (*P*<0.05). However, the DKD group showed significant decreases in education level above high school, moderate physical activity, LDL, and eGFR (*P*<0.05). Notably, the DKD group exhibited higher WWI levels than the non-DKD group (11.75 ± 0.03 vs. 11.53 ± 0.02, *P*<0.001).

**Table 1:** Basic characteristics of T2DM participants, weighted.

	Overall (N=5028)	Non-DKD (N=3087)	DKD (N=1941)	*P* value
Age (years)	58.89±0.29	56.26±0.32	63.72 ±0.44	<0.001
Male gender, % (SE)	53.33 (1.05)	53.54 (1.52)	52.96 (1.70)	0.816
Race, % (SE)				0.760
Mexican American	10.11 (1.12)	10.25 (1.18)	9.85 (1.17)	
Non-Hispanic Black	14.25 (1.07)	14.13 (1.15)	14.48 (1.15)	
Non-Hispanic White	60.44 (1.84)	60.26 (2.03)	60.75 (1.93)	
Other Hispanic	6.03 (0.63)	6.32 (0.68)	5.50 (0.71)	
Other Races	9.17 (0.68)	9.04 (0.80)	9.42 (0.91)	
Annual household income (under $20,000), % (SE)	17.19 (0.76)	15.00 (0.95)	21.24 (1.08)	<0.001
Education level (above high school), % (SE)	52.59 (1.07)	55.04 (1.56)	48.06 (1.79)	0.008
Moderate physical activity, % (SE)	35.63 (1.05)	38.04 (1.41)	31.19 (1.26)	<0.001
Smokers, % (SE)	50.86 (0.96)	50.02 (1.38)	52.40 (1.62)	0.307
Hypertension, % (SE)	64.17 (1.03)	58.62 (1.22)	74.42 (1.46)	<0.001
Cardiovascular disease, % (SE)	22.78 (0.85)	17.64 (1.08)	32.27 (1.51)	<0.001
BMI (kg/m^2^)	32.98 (0.18)	32.91 (0.23)	33.12 (0.25)	0.070
Weight (kg)	93.10 ±0.52	93.34±0.64	92.67±0.87	0.531
WC (cm)	111.29±0.38	110.78±0.48	112.23 ±0.54	0.041
ALT (U/L)	27.99±0.55	28.38±0.54	27.29±1.12	0.370
AST (U/L)	26.69 ±0.38	26.57±0.43	26.90±0.60	0.630
GGT (U/L)	35.59±0.77	34.00 ±0.84	38.55±1.51	0.010
Glycohemoglobin (%)	7.25 ±0.03	7.08 ±0.04	7.55±0.05	<0.001
FPG (mmol/L)	8.72±0.09	8.44 ±0.10	9.23±0.13	<0.001
TG (mmol/L)	1.85±0.05	1.75 ±0.06	2.03 ±0.07	0.001
TC (mmol/L)	4.78 ±0.02	4.80±0.03	4.74 ±0.03	0.258
HDL-c (mmol/L)	1.21 ±0.01	1.22 ±0.01	1.21±0.01	0.620
LDL-c (mmol/L)	2.69 ±0.03	2.73±0.04	2.61 ±0.04	0.028
BUN (mmol/L)	5.69±0.06	5.00±0.05	6.96±0.12	<0.001
SUA (umol/L)	339.27 ±2.18	324.97 ±2.23	365.66 ±3.30	<0.001
Scr (umol/L)	83.97±0.88	73.42±0.46	103.45±2.26	<0.001
eGFR (ml/min/1.73 m^2^)	85.12 ±0.44	92.96 ±0.40	70.64 ±0.97	<0.001
urinary albumin (mg/L)	117.63±9.87	11.71±0.23	313.08±27.59	<0.001
urinary creatinine (mg/dL)	117.33±1.43	119.25±2.00	113.80 ±2.08	0.075
UACR (mg/g)	118.23±9.43	10.13 ±0.14	317.71 ±26.78	<0.001
WWI index	11.61±0.02	11.53 ±0.02	11.75±0.03	<0.001

BMI, body mass index; WC, waist circumference;ALT, alanine transaminase; AST, aspartate transaminase; GGT, gamma-glutamyl transferase; FPG, fasting plasma glucose; TG, triglyceride; TC, total cholesterol; HDL-c, high-density lipoprotein cholesterol; LDL-c, low-density lipoprotein cholesterol; BUN, blood urea nitrogen; SUA, serum uric acid; Scr, serum creatinine; eGFR, estimated glomerular filtration rate; UACR, urinary albumin to urinary creatinine ratio; WWI index, weight-adjusted-waist index.

### 3.2 Clinical features of T2DM participants according to WWI index quantiles

The T2DM participants were divided into four groups based on their WWI levels: quartile I, quartile II, quartile III, and quartile IV, as detailed in **Table 2**. When compared to the quartile I-WWI group, the quartile II-WWI, quartile III-WWI, and quartile IV-WWI groups showed significant increases in age, annual household income under $20,000, prevalence of hypertension, cardiovascular disease, BMI, Weight, WC, BUN, SUA, urinary albumin, and UACR (*P*<0.01). Additionally, male, education level above high school, moderate physical activity, FPG, HDL-c, eGFR, and urinary creatinine exhibited significant decreases (*P*<0.05). There were also significant differences in the distribution of race among the various quartile groups (*P*<0.001). No differences in smokers, ALT, AST, GGT, Glycohemoglobin, TG, TC, LDL-c, and Scr were observed between groups. Importantly, as the WWI levels increased progressively, both the prevalence of albuminuria, low-eGFR, and DKD also showed a gradual increase (albuminuria: 20.14% vs. 26.00% vs. 26.58% vs. 30.77%, *P*<0.001; low-eGFR: 10.29% vs. 12.26% vs. 20.52% vs. 22.76%, *P*<0.001; DKD: 26.76% vs. 32.63% vs. 39.06% vs. 42.96%, *P*<0.001).

**Table 2:** Clinical features of T2DM participants according to WWI index quantiles, weighted.

	Quartile 1	Quartile 2	Quartile 3	Quartile 4	*P* value
Age (years)	53.71±0.48	58.14 ±0.46	61.13 ±0.49	63.08 ±0.48	<0.001
Male gender, % (SE)	68.85 (2.08)	61.94 (2.04)	51.99 (2.24)	29.11 (2.01)	<0.001
Race, % (SE)					<0.001
Mexican American	8.20 (1.12)	11.60 (1.45)	10.47 (1.52)	10.36 (1.24)	
Non-Hispanic Black	19.30 (1.60)	15.06 (1.57)	12.32 (1.23)	9.81 (1.12)	
Non-Hispanic White	56.25 (2.48)	56.79 (2.39)	63.11 (2.51)	66.00 (2.07)	
Other Hispanic	5.73 (0.71)	6.18 (0.85)	6.37 (0.92)	5.88 (0.70)	
Other Races	10.52 (1.10)	10.36 (1.21)	7.73 (0.90)	7.95 (1.19)	
Annual household income (under $20,000), % (SE)	12.02 (0.98)	16.13 (1.18)	17.53 (1.31)	23.56 (1.65)	<0.001
Education level (above high school), % (SE)	58.92 (2.14)	52.94 (2.04)	54.45 (1.81)	43.46 (2.18)	<0.001
Moderate physical activity, % (SE)	41.58 (1.74)	35.91 (1.88)	33.98 (2.16)	30.48 (2.16)	0.001
Smokers, % (SE)	48.20 (2.16)	51.06 (1.99)	52.73 (1.87)	51.71 (2.00)	0.421
Hypertension, % (SE)	54.01 (2.28)	63.16 (1.95)	68.77 (1.71)	71.75 (1.87)	<0.001
Cardiovascular disease, % (SE)	15.25 (1.57)	21.39 (1.74)	25.16 (1.43)	30.06 (1.75)	<0.001
BMI (kg/m^2^)	29.68 (0.28)	32.07 (0.22)	34.38 (0.22)	36.12 (0.31)	<0.001
Weight (kg)	88.52±0.85	92.66 ±0.73	96.62 ±0.86	95.09 ±0.99	<0.001
WC (cm)	100.26 ±0.54	108.92 ±0.43	115.62 ±0.49	121.44 ±0.63	<0.001
ALT (U/L)	27.21±0.66	29.59±0.98	28.11±0.81	27.16±1.49	0.118
AST (U/L)	25.65±0.49	27.48±0.71	26.77±0.66	26.96 ±0.95	0.141
GGT (U/L)	33.75±1.39	35.96±1.55	35.34±1.35	37.51±1.54	0.328
Glycohemoglobin (%)	7.25±0.07	7.26 ±0.07	7.15 ±0.06	7.33 ±0.06	0.321
FPG (mmol/L)	8.93±0.19	8.83 ±0.15	8.38 ±0.13	8.72 ±0.18	0.036
TG (mmol/L)	1.69±0.08	1.99 ±0.11	1.82 ±0.08	1.91 ±0.08	0.058
TC (mmol/L)	4.80±0.04	4.79 ±0.06	4.71 ±0.05	4.81 ±0.05	0.474
HDL-c (mmol/L)	1.27±0.02	1.17 ±0.01	1.20 ±0.02	1.21 ±0.02	<0.001
LDL-c (mmol/L)	2.75±0.05	2.72 ±0.06	2.67 ±0.06	2.61 ±0.07	0.367
BUN (mmol/L)	5.48±0.10	5.48±0.08	5.80±0.11	5.99±0.10	<0.001
SUA (umol/L)	322.47 ±4.02	336.78 ±3.68	345.78 ±3.14	353.75 ±4.04	<0.001
Scr (umol/L)	85.37 ±2.10	84.00±1.44	84.61±1.44	81.78 ±1.26	0.312
eGFR (ml/min/1.73 m^2^)	90.04±0.82	87.36 ±0.79	82.51±0.97	80.09 ±0.88	<0.001
Urinary albumin(mg/L)	82.18±11.10	182.31 ±31.57	109.14 ±16.10	100.67 ±11.64	0.008
Urinary creatinine(mg/dL)	127.34 ±3.45	122.52 ±3.29	112.54 ±2.59	105.95 ±2.25	<0.001
UACR (mg/g)	76.01 ±10.79	155.07 ±23.98	133.75±22.68	112.58 ±14.11	0.002
Albuminuria, % (SE)	20.14 (1.64)	26.00 (1.63)	26.58 (1.74)	30.77 (1.77)	<0.001
Low-eGFR, % (SE)	10.29 (1.18)	12.26 (1.09)	20.52 (1.37)	22.76 (1.38)	<0.001
DKD, % (SE)	26.76 (1.97)	32.63 (1.84)	39.06 (1.95)	42.96 (1.83)	<0.001

### 3.3 Correlation of WWI index with clinical parameters

In the weighted Pearson correlation analysis, WWI index showed significant associations with several variables (**Table 3**). Specifically, WWI index was positively correlated with age, annual household income under $20,000, hypertension, cardiovascular disease, BMI, weight, WC, BUN, SUA, albuminuria, low-eGFR, DKD (*P*<0.05). Conversely, WWI index was negatively correlated with male, education level above high school, moderate physical activity, TC, HDL-c, LDL-c, eGFR, and urinary creatinine (*P*<0.05). After adjusting for age, gender, and race, the WWI index was still positively correlated with albuminuria, low-eGFR, DKD (*P*<0.05).

**Table 3:** Pearson correlation analysis results of WWI index with other parameters in T2DM population, weighted.

	Non-adjusted	Adjusted for age, gender, and race
Age (years)	0.275**	–
Male	-0.298**	–
Race	-0.015	–
Under $20,000	0.114**	0.073**
Above high school	-0.108**	-0.080**
Moderate physical activity	-0.065**	-0.008
Smokers	-0.01	0.052**
Hypertension	0.127**	0.068**
Cardiovascular disease	0.129**	0.104**
BMI (kg/m^2^)	0.333**	0.419**
Weight (kg)	0.096**	0.289**
WC (cm)	0.503**	0.627**
ALT (U/L)	-0.015	0.065**
AST (U/L)	0.004	0.02
GGT (U/L)	-0.002	0.050*
Glycohemoglobin (%)	-0.008	0.056**
FPG (mmol/L)	-0.032	0.033
TG (mmol/L)	0.022	0.183**
TC (mmol/L)	-0.035*	-0.040*
HDL-c (mmol/L)	-0.079**	-0.228**
LDL-c (mmol/L)	-0.068**	-0.03
BUN (mmol/L)	0.119**	0.058**
SUA (umol/L)	0.074**	0.143**
Scr (umol/L)	-0.018	-0.024
eGFR (ml/min/1.73 m^2^)	-0.177**	-0.017
Urinary albumin (mg/L)	-0.004	0.008
Urinary creatinine (mg/dL)	-0.134**	-0.037
UACR (mg/g)	0.013	0.022
Albuminuria	0.083**	0.097**
Low-eGFR	0.134**	0.047*
DKD	0.126**	0.089**

* **P**<0.05 ** **P**<0.01.

### 3.4 Associations between WWI index and DKD, low-eGFR, and albuminuria risk

**Figure 1:**
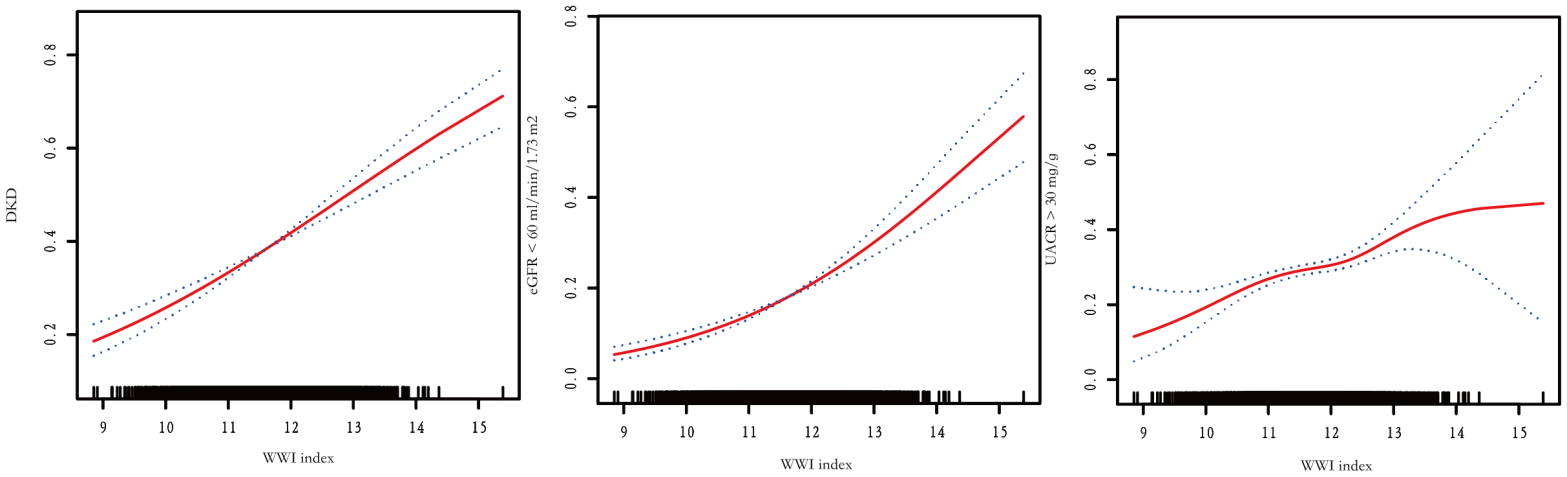
Smooth curve fitting results of WWI index and DKD, low-eGFR, and albuminuria risk.

The association between WWI index and DKD risk was presented in **Table 4**. The unadjusted model revealed a positive association between WWI index and an increased risk of DKD (OR=1.44, 95%CI:1.33-1.56, *P*< 0.001). This association remained statistically significant after adjusting for age, gender, and race (OR=1.36, 95%CI:1.24-1.48, *P*< 0.001). After additional adjustment for multiple covariates, the results indicated a 32% increased risk of DKD per unit increase in WWI index (OR=1.32, 95%CI:1.12-1.56, *P*<0.001). Similarly, we also observed a significant association between the WWI index and low-eGFR, and albuminuria after adjusting covariates (low-eGFR: OR=1.24, 95%CI:1.00-1.52, *P*=0.049; albuminuria: OR=1.37, 95%CI:1.17-1.60, *P*< 0.001) (**Data Sheet 1**). When classifying WWI index into quartiles, we found that higher WWI quartiles were associated with a higher prevalence of DKD compared to the lowest quartiles in the fully adjusted model (OR=1.47, 95%CI:1.06-2.04, *P*=0.021). Additionally, the results of the smooth curve fitting analysis confirmed a positive correlation between WWI index and the risk of DKD, low-eGFR, and albuminuria risk (**Figure 1**). No significant threshold effect was observed in DKD.

**Table 4:** The relationship between WWI index and DKD risk.

DKD	OR (95%CI), *P* value		
	Non-adjusted model 1	Adjusted model 2	Adjusted model 3
Continuous			
Weight	1.00 (1.00, 1.00), 0.251	1.00 (1.00, 1.00), 0.163	1.00 (0.99, 1.00), 0.397
WC	1.00 (1.00, 1.01), 0.010	1.01 (1.00, 1.01), <0.001	1.00 (0.99, 1.01), 0.445
WWI index	1.44 (1.33, 1.56), <0.001	1.36 (1.24, 1.48), <0.001	1.32 (1.12, 1.56), <0.001
Categories			
Quartile 1	1.00	1.00	1.00
Quartile 2	1.19 (1.01, 1.40), 0.043	1.08 (0.91, 1.28), 0.393	1.21(0.91, 1.62), 0.196
Quartile 3	1.41 (1.20, 1.67), <0.001	1.24 (1.04, 1.47), 0.016	1.31 (0.97, 1.76), 0.078
Quartile 4	1.89 (1.61, 2.22), <0.001	1.65 (1.38, 1.97), <0.001	1.47 (1.06, 2.04), 0.021
***P*** for trend	<0.001	<0.001	0.021

OR, odds ratio. 95% CI, 95% confidence interval. Adjusted model 2: age, gender, and race were adjusted. Adjusted model 3: additionally adjusted for annual household income, education level, moderate physical activity, smokers, hypertension, cardiovascular disease, BMI, ALT, AST, GGT, FPG, glycohemoglobin, TG, TC, HDL-c, LDL-c, BUN, SUA, and Scr.

### 3.5 Multivariate logistic regression model of DKD

A multivariate logistic regression model was performed using WWI index, age, gender, race, annual household income, education level, moderate physical activity, smokers, hypertension, cardiovascular disease, BMI, ALT, AST, GGT, FPG, glycohemoglobin, TG, TC, HDL-c, LDL-c, BUN, SUA, and Scr as independent variables, and DKD as dependent variables. The results showed that WWI index, age, gender, race, smokers, hypertension, GGT, glycohemoglobin, BUN, SUA, and Scr were more strongly associated with DKD risk (*P*< 0.05) (**Table 5**).

**Table 5:** Multivariate logistic regression model of DKD.

	OR	95%CI lower	95%CI upper	*P* value
WWI index	1.325	1.123	1.563	<0.001
Age (years)	1.015	1.004	1.025	0.007
Male	0.459	0.353	0.598	<0.001
Race (vs. Mexican American)				
Non-Hispanic Black	0.443	0.312	0.629	<0.001
Non-Hispanic White	0.650	0.479	0.881	0.005
Other Hispanic	0.645	0.444	0.937	0.021
Other Races	0.858	0.584	1.263	0.438
Under $20,000	1.112	0.879	1.406	0.378
Above high school	0.837	0.678	1.034	0.099
Moderate physical activity	0.922	0.742	1.146	0.466
Smokers	1.232	1.003	1.512	0.047
Hypertension	1.260	1.012	1.570	0.039
Cardiovascular disease	1.186	0.937	1.503	0.157
BMI (kg/m^2^)	0.998	0.981	1.015	0.809
ALT (U/L)	0.995	0.985	1.004	0.256
AST (U/L)	1.001	0.994	1.008	0.761
GGT (U/L)	1.004	1.002	1.006	0.001
FPG (mmol/L)	1.012	0.965	1.061	0.630
Glycohemoglobin (%)	1.329	1.212	1.457	<0.001
TG (mmol/L)	1.017	0.949	1.091	0.630
TC (mmol/L)	0.916	0.646	1.299	0.624
HDL-c (mmol/L)	1.095	0.772	1.553	0.612
LDL-c (mmol/L)	1.092	0.770	1.549	0.620
BUN (mmol/L)	1.092	1.025	1.164	0.006
SUA (umol/L)	1.002	1.000	1.003	0.017
Scr (umol/L)	1.046	1.038	1.053	<0.001

### 3.6 ROC analysis

**Figure 2:**
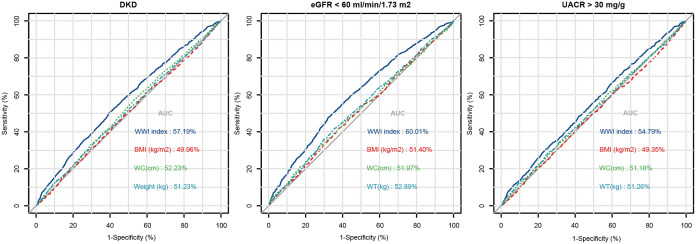
ROC curve analysis of WWI index in assessing DKD, low-eGFR, and albuminuria risk.

**Figure 2** illustrates the evaluation of WWI index, Weight, WC, and BMI, in terms of their predictive power for low-eGFR, albuminuria, and DKD risk based on the ROC analysis. In terms of DKD risk, the corresponding AUC values are WWI index (57.19%), Weight (51.23%), WC (52.23%), and BMI (49.96%). The corresponding AUC values in low-eGFR risk are as follows: WWI index (60.01%), Weight (52.89%), WC (51.97%), and BMI (51.40%). As for albuminuria risk, the corresponding AUC values are WWI index (54.79%), Weight (51.26%), WC (51.18%), and BMI (49.35%).

### 3.7 Subgroup analyses

**Figure 3:**
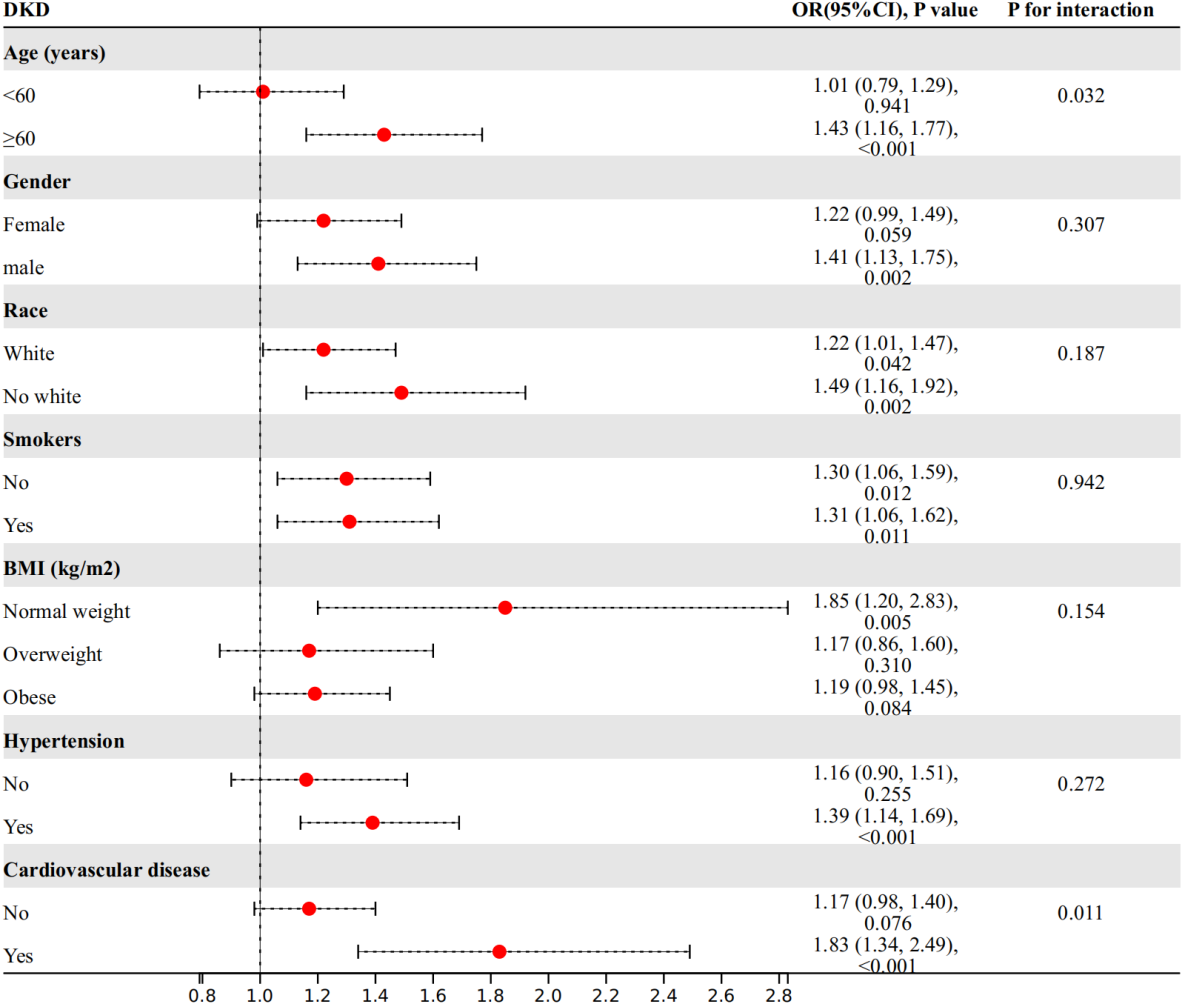
Subgroup analysis.

We conducted further investigations into the relationship between WWI index and DKD risk in different subgroups based on age, gender, race, smoking status, BMI, hypertension, and cardiovascular disease (**Figure 3**). Our findings showed a significant interaction between age, cardiovascular disease, and WWI index in relation to DKD risk (*P* for interaction<0.05). Specifically, WWI index had a stronger association with the risk of DKD in the elderly (age≥60 years) (OR=1.43, 95%CI:1.16-1.77, *P*<​0.001 vs. OR=1.01, 95%CI:0.79-1.29, *P*=0.941) and cardiovascular disease (OR=1.83, 95%CI:1.34-2.49, *P*<​0.001 vs. OR=1.17, 95%CI:0.98-1.40, *P*= 0.076) population. No significant interactions were observed in the other subgroups (*P* for interaction> 0.05).

## 4 Discussion

To our knowledge, this is the first study based on the T2DM population to explore the relationship between the WWI index and the risk of DKD. Our study shows that the WWI index is independently associated with the risk of DKD, and this association is more pronounced in the elderly and cardiovascular disease population. The WWI index is a highly potential and simple epidemiological tool to quantify the role of abdominal obesity in the risk of DKD.

While some evidence suggests that traditional anthropometric measures are potentially linked to DKD in epidemiological studies, the obesity paradox still exists. This is partly due to the complex correlations among different anthropometric measures, making it difficult to identify biologically driven disease risk (14). Therefore, it is particularly important to accurately distinguish between lean body weight and fat mass. In addition, compared to emerging obesity indices that rely on relatively complex empirical mathematical models, WWI index has the advantage of being very easy to calculate, making it feasible to conduct routine checks in the general population. Previous study also indicated that WWI index could welly differentiate between fat and muscle mass, positively correlating with fat mass and negatively correlating with muscle mass (15, 16). In a longitudinal analysis about 40 months, CT data from 1,946 participants showed an increase in abdominal fat measures and a decrease in muscle measures, along with an increase in WWI index (17). Additionally, one cohort study has also shown that WWI index exhibits higher excellence compared to BMI, WC, WHtR, and ABSI which measured by dividing WC by an allometric regression of weight and height (18, 19). Therefore, as a measure of body composition, WWI shows promise for further exploration due to its simplicity in calculation and good performance in differentiating body composition and predicting disease risk (7). Previous studies have also suggested the role of the WWI index in albuminuria risk in the general population (7). Our study in the T2DM population confirmed this, and we also found that the WWI index is also associated with low eGFR. Combining the two, we observed a clear linear relationship between the WWI index and the risk of DKD. It is noteworthy that through subgroup analysis, we have found that in the high-risk population with renal impairment, specifically in the elderly and individuals with cardiovascular diseases, this relationship is more pronounced. Therefore, it is important to pay closer attention to changes in the WWI index levels in these specific populations.

Some potential pathological and physiological mechanisms can explain this relationship. The intricate connection between abdominal obesity and DKD involves a complex interplay of metabolic, inflammatory, and hemodynamic factors. Abdominal obesity is linked to the production of inflammatory cytokines and adipokines, such as TNF-α, IL-6, adiponectin and leptin, which can be involved in kidney damage (20). For example, adiponectin has been shown to regulate inflammation and oxidative stress, thereby safeguarding the kidneys (21). Conversely, a deficiency in adiponectin can lead to an increase in reactive oxygen species, which may have detrimental effects on the kidneys (21). On the other hand, obesity-induced immunosuppression leads to urinary tract infections, which in turn exacerbate renal dysfunction (22). Abdominal obesity is associated with changes in renal hemodynamics, such as elevated glomerular filtration rate and intraglomerular pressure, which can further drive the development and progression of DKD (23, 24). Furthermore, abdominal obesity is often associated with other metabolic abnormalities, such as dyslipidemia and hypertension, which further contribute to the development and progression of diabetic kidney disease (25, 26). Importantly, obesity is closely linked to insulin resistance, which increases sodium reabsorption and glomerular hyperfiltration, thus enhancing renal damage (27, 28). Additionally, insulin resistance accelerates CKD progression through chronic inflammation and oxidative stress-induced damage to podocytes and the basement membrane (27, 28).

However, it is important to recognize the limitations of this study. Firstly, the cross-sectional design is insufficient to establish a causal relationship between WWI index and the risk of DKD. Cohort studies and intervention trials are needed to determine whether there is a causal relationship between the two. Secondly, some potential confounding factors, such as metabolic syndrome and non-alcoholic fatty liver disease, were not included in the study. Lastly, the sample used in our study is from the US population, and the generalizability of our findings needs further validation in other populations.

## 5 Conclusion

Our study suggests that higher WWI index is associated with an increased risk of DKD. The predictive ability of WWI index for DKD was stronger compared to BMI and WC, but our results need further validation through more research.

## Data availability statement

Publicly available datasets were analyzed in this study. This data can be found here: https://www.cdc.gov/nchs/nhanes/.

## Ethics statement

The studies involving humans were approved by the Ethics Review Board of the National Center for Health Statistics. The studies were conducted in accordance with the local legislation and institutional requirements. The participants provided their written informed consent to participate in this study.

## Author contributions

ZW: Writing – review & editing, Writing – original draft. XS: Writing – original draft. WX: Writing – original draft. BX: Writing – original draft. SZ: Writing – original draft. QY: Writing – review & editing, Writing – original draft, Funding acquisition.

## Supplementary material

DATA SHEET 1 The relationship between WWI index and low-eGFR, albuminuria risk.
