# Supplementary material for: The relationship between weight-adjusted-waist index and diabetic kidney disease in patients with type 2 diabetes mellitus
Source: Front Endocrinol (Lausanne). 2024 Mar 15;15:1345411. doi: 10.3389/fendo.2024.1345411 (PMC10978751; doi:10.3389/fendo.2024.1345411)
Supplement: DATA SHEET 1 — The relationship between WWI index and low-eGFR, albuminuria risk. [file DataSheet_1.docx]

| The relationship between WWI index and Albuminuria risk | | | |
| --- | --- | --- | --- |
| UACR > 30 mg/g | OR (95%CI), ***P*** value | | |
|  | Non-adjusted model 1 | Adjusted model 2 | Adjusted model 3 |
| Continuous |  |  |  |
| Weight | 1.00 (1.00, 1.00), 0.263 | 1.00 (1.00, 1.00), 0.889 | 1.00 (0.99, 1.00), 0.453 |
| WC | 1.00 (1.00, 1.01), 0.210 | 1.00 (1.00, 1.01), 0.025 | 1.01 (1.00, 1.01), 0.260 |
| WWI index | 1.29 (1.19, 1.40), <0.001 | 1.34 (1.22, 1.47), <0.001 | 1.37 (1.17, 1.60), <0.001 |
| Categories |  |  |  |
| Tertile 1 | 1.00 | 1.00 | 1.000 |
| Tertile 2 | 1.21 (1.01, 1.44), 0.035 | 1.18 (0.99, 1.41), 0.071 | 1.28 (0.96, 1.70), 0.087 |
| Tertile 3 | 1.22 (1.02, 1.45), 0.028 | 1.21 (1.01, 1.45), 0.044 | 1.33 (0.99, 1.78), 0.057 |
| Tertile 4 | 1.58 (1.33, 1.88), <0.001 | 1.65 (1.36, 1.99), <0.001 | 1.62 (1.18, 2.22), 0.003 |
| ***P*** for trend | <0.001 | <0.001 | 0.004 |
| OR: odds ratio.  95% CI: 95% confidence interval  Adjusted model 2: age, gender, and race were adjusted.  Adjusted model 3: additionally adjusted for annual household income, education level, moderate physical activity, smokers, hypertension, cardiovascular disease, BMI, ALT, AST, GGT, FPG, glycohemoglobin, TG, TC, HDL-c, LDL-c, BUN, SUA, and Scr. | | | |

| The relationship between WWI index and low-eGFR risk | | | |
| --- | --- | --- | --- |
| eGFR < 60 mL/min/1.73m^2^ | OR (95%CI), ***P*** value | | |
|  | Non-adjusted model 1 | Adjusted model 2 | Adjusted model 3 |
| Continuous |  |  |  |
| Weight | 1.00 (0.99, 1.00), 0.005 | 1.00 (1.00, 1.01), 0.418 | 0.98 (0.96, 1.00), 0.033 |
| WC | 1.00 (1.00, 1.01), 0.108 | 1.01 (1.00, 1.01), 0.005 | 1.00 (0.98, 1.02), 0.887 |
| WWI index | 1.63 (1.47, 1.81), <0.001 | 1.36 (1.21, 1.53), <0.001 | 1.24 (1.00, 1.52), 0.049 |
| Categories |  |  |  |
| Tertile 1 | 1.00 | 1.00 | 1.00 |
| Tertile 2 | 1.26 (1.00, 1.58), 0.048 | 1.01 (0.79, 1.29), 0.932 | 1.32 (0.88, 1.97), 0.177 |
| Tertile 3 | 1.78 (1.43, 2.21), <0.001 | 1.29 (1.02, 1.64), 0.032 | 1.35 (0.90, 2.01), 0.147 |
| Tertile 4 | 2.41 (1.95, 2.97), <0.001 | 1.65 (1.30, 2.08), <0.001 | 1.56 (1.02, 2.38), 0.040 |
| ***P*** for trend | <0.001 | <0.001 | 0.056 |
| OR: odds ratio.  95% CI: 95% confidence interval  Adjusted model 2: age, gender, and race were adjusted.  Adjusted model 3: additionally adjusted for annual household income, education level, moderate physical activity, smokers, hypertension, cardiovascular disease, BMI, ALT, AST, GGT, FPG, glycohemoglobin, TG, TC, HDL-c, LDL-c, BUN, SUA, and Scr. | | | |
